# Mobile app for pelvic floor muscle training for urinary incontinence during the coronavirus disease 2019 pandemic: clinical trial

**DOI:** 10.1590/1806-9282.20231073

**Published:** 2024-04-22

**Authors:** Camila Carvalho de Araujo, Luiz Gustavo Oliveira Brito, Andrea Marques, Marcela Bardin, Cássia Raquel Teatin Juliato

**Affiliations:** 1 Universidade Estadual de Campinas, School of Medical Sciences, Department of Obstetrics and Gynecology – Campinas (SP), Brazil.

**Keywords:** Adherence, COVID-19, Mobile app, Pelvic floor, Urinary incontinence

## Abstract

**OBJECTIVE:**

The objective of this study was to evaluate the effects of home-based pelvic floor muscle training in women with urinary incontinence, addressing the difficulties arising from social isolation due to the coronavirus disease 2019 pandemic by utilizing a specialized mobile app.

**METHODS:**

This randomized, single-group clinical trial aimed to assess the efficacy of pelvic floor muscle training guided by a mobile app (Diario Saúde) in women with stress urinary incontinence. Participants were instructed via telephone to engage in pelvic floor muscle training exercises twice a day for 30 days. Pre- and post-treatment, participants completed validated questionnaires regarding urinary symptoms and quality of life through telephone interviews. Additionally, treatment adherence was evaluated.

**RESULTS:**

A total of 156 women were enrolled in the study, with a mean age of 49.3±14.2 years. Significant improvements in urinary incontinence symptoms and quality of life were observed following pelvic floor muscle training guided by the mobile app (p<0.001). Notably, 74.3% of the participants reported performing the exercises with appropriate frequency. Of the participants, 62% reported either complete or substantial improvement in urinary symptoms post-treatment.

**CONCLUSION:**

This study revealed notable enhancements in stress urinary incontinence, urinary storage, and overall quality of life subsequent to pelvic floor muscle training guided by a mobile app, particularly during the coronavirus disease 2019 pandemic. The mobile app demonstrated robust acceptance and adherence among women experiencing urinary incontinence.

## BACKGROUND

Urinary incontinence (UI) represents a substantial global health concern for women, exerting adverse effects on their quality of life and contributing to increased morbidity and mortality rates^1^. Pelvic floor muscle training (PFMT) stands as the primary conservative approach for managing UI in women^2^, yielding favorable outcomes. Nevertheless, adhering to PFMT presents complexities, requiring modifications in behavior and active patient engagement^3^.

The onset of the coronavirus disease (COVID-19) pandemic demanded both patients and healthcare providers adapt to new norms, emphasizing social isolation and rigorous sanitation protocols^4^. As a result, addressing non-urgent conditions like UI became challenging due to pandemic-induced restrictions^4^.

Telemedicine emerged as an innovative solution during this period, gaining widespread acceptance^4,5^. Although mobile apps for PFMT had been explored previously, the effectiveness, acceptance, and adherence of women with UI to a structured app-based PFMT regimen took on heightened significance within the context of the pandemic.

Adherence to PFMT exercises remains crucial for sustaining long-term effectiveness and preventing UI recurrence^6^. A recent systematic review confirmed the safety and efficacy of home device apps^7^. This study aimed to evaluate the effects of home-based PFMT in women with UI, addressing the difficulties arising from social isolation due to the COVID-19 pandemic by utilizing a specialized mobile app Diário Saúde^®8^.

## DESIGN AND SETTING

This randomized, single-group, clinical trial study was conducted from December 2020 to March 2022 at the University of Campinas, Brazil. The study was approved by the local research ethics board (CAAE: 36515520.0.0000.5404) and registered at the Brazilian Clinical Trials Registry (RBR-7k2hpj4). Informed consent was obtained from all participants through a recorded telephone call after the agreement of the participant, who also provided a digital signature through the consent term sent by email.

### Participants

We included women aged 18 years or older, with complaints of stress urinary incontinence (SUI) or mixed UI with stress predominance according to the International Continence Society criteria^9^ lasting for at least 4 weeks, who had an Android mobile phone, were able to read, agreed to answer the questionnaires through telephone contact, and performed PFMT using the mobile app Diário Saúde^®10^. We excluded women who were currently pregnant or using medication for UI, had any prior UI treatment, had recurrent urinary tract infection, had a history of neurological diseases, and were <1 year postpartum.

### Procedure and intervention

Recruitment of participants for the study was initiated through outreach on social networks, inviting interested women to connect with the principal researcher (a qualified physiotherapist) for an initial screening interview aimed at confirming eligibility criteria. Subsequently, comprehensive interviews were conducted with all participants, during which the exercise program was introduced and explained via telephone communication.

Participants underwent a baseline assessment encompassing sociodemographic and gynecological information, details about COVID-19 testing and outcomes, and any changes in urinary symptoms when COVID-19 was confirmed and answered the validated Portuguese versions of the following questionnaires: Questionnaire for Urinary Incontinence Diagnosis (QUID), International Consultation on Incontinence Questionnaire-Short Form (ICIQ-SF), International Consultation on Incontinence Questionnaire-Overactive Bladder (ICIQ-OAB), and Incontinence Quality of Life Questionnaire (I-QOL)^10–13^. Participants received thorough education regarding the role of pelvic floor muscles, accurate perception of their activation, and the correct techniques for contraction and relaxation. Subsequently, participants were guided to install the dedicated mobile app.

The home-based training protocol was based on the regimen outlined by Araujo et al.^9^ involving exercises lasting around 5 min each, to be performed twice daily (in sitting, lying, or standing position) over a span of 30 days.

A post-treatment assessment was conducted by the researcher 30–45 days after the exercise period's conclusion, utilizing the same baseline assessment tools. Additionally, participants were asked about their satisfaction with the treatment intervention and to rate their satisfaction with the treatment. Finally, adherence to the treatment regimen was measured through the question, "How often did you adhere to the prescribed exercises?"

#### Diário Saúde^®^ app

The Diário Saúde^®^ app uses a visual stimulus similar to electromyography as a guide for PFMT contraction, which shows when the participant is supposed to contract and sustain or contract and relax, along with the duration of contraction and the number of repetitions. The music rhythm is synchronized with the duration of the contractions, and the volume changes during the contraction or relaxation of pelvic floor muscle visual stimuli^8^. Importantly, the app also offers twice-a-day reminders to encourage practicing PFMT.

### Outcomes

The main objective of this study was to assess the enhancement in urinary symptoms, urinary storage, and quality of life. The secondary objective was to encompass the assessment of both adherence to and satisfaction with the intervention among women experiencing UI.

### Statistical analysis

The sample's characteristics were depicted by presenting frequency tables for categorical variables with absolute frequency (n) and percentage (%) values. Descriptive statistics, including mean values and standard deviations, were employed to portray the numerical variables. To compare categorical variables before and after the intervention, the McNemar test was applied for related samples involving two categories. In cases where numerical variables lacked a normal distribution, the Wilcoxon test was employed to compare related samples before and after the intervention. The threshold for statistical significance was set at 5%, denoted as p<0.05^14^.

## RESULTS

**Figure 1:**
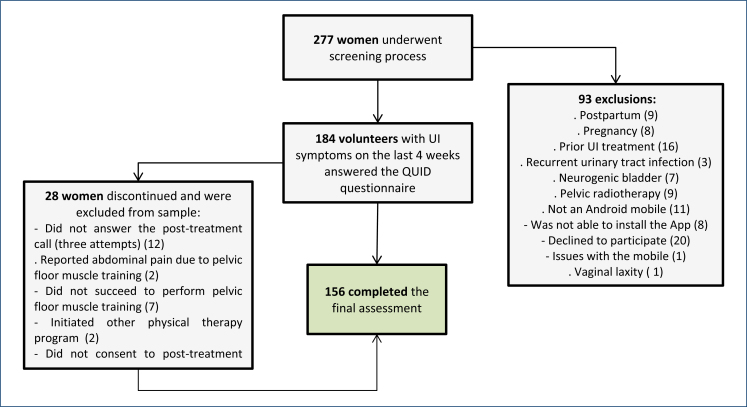
Participant flowchart and drop-out. UI: Urinary incontinence; PFMT: pelvic floor muscle training.

A total of 156 women with complaints of SUI or mixed UI with stress predominance were included in the study (Figure 1). The mean age of the women was 49.3±14.2 years. Among the women affected by UI, 23.7% had tested positive for COVID-19, and of those, half noted a deterioration in UI symptoms subsequent to the infection (Table 1).

**Table 1:** Characteristics of UI women included the study.

Variables		Total (n=156)
Age, mean (SD)		49.3 (14.2)
Marital status, n (%)		
	Single	28 (18)
	Married/living with a partner	86 (55.1)
	separated/divorced/widowed	42 (26.9)
Ethnicity, n (%)		
	White	108 (69.2)
	Others	48 (30.8)
BMI, mean (SD)		29.1 (6.4)
Number of pregnancies, mean (SD); n=140		
	Vaginal delivery	1.5 (1.2)
	Cesarean	0.8 (0.8)
	Abortion	0.5 (0.9)
Pregnancies, mean (SD); n=140		
	Vaginal	106 (75.7)
	Cesarean	78 (55.7)
	Abortion	44 (31.4)
Weight birth (grams), mean (SD); n=136		3385 (699)
Education (years), mean (SD)		12.3 (3.8)
Comorbidities, n (%)		
	Arterial hypertension	50 (32)
	Diabetes mellitus	29 (18.6)
	Chronic obstructive pulmonary disease	7 (4.5)
Smoking, n (%)		18 (11.5)
Menopause, n (%)		77 (49.4)
Physical activity, n (%)		58 (37.2)
Intestinal constipation, n (%)		65 (41.7)
Onset of UI symptoms, n (%)		
	During pregnancy	71 (45.5)
	After menopause	28 (18)
	Don't know	57 (36.5)
Positive COVID test, n (%)		37 (23.7)
Worsening of IU symptoms after COVID, n (%); n=37		19 (51.3)

UI: urinary incontinence; SD: standard deviation; N: absolute frequency; BMI: body mass index.

The majority of participants in our study exhibited severe to very severe SUI, as indicated by ICIQ-SF scores ranging from 5 to 21, with a median of 14.4 (±4) and a mean of 15, accompanied by interquartile 25 and interquartile 75 values of 12 and 18, respectively. Significant enhancements were observed in UI symptoms assessed by QUID and ICIQ-SF following the mobile app-based home treatment (p<0.001). Furthermore, improvements were evident in urinary storage symptoms post-treatment (7.3 vs. 6.3, p<0.001). Evaluation of quality of life indicated overall improvement post-treatment, encompassing aspects such as general well-being, avoidance or limitation of behaviors, psychological impact, and social discomfort (p<0.001, Table 2).

**Table 2:** Urinary symptoms and quality of life in women with urinary incontinence before and after treatment with an app-guided pelvic floor muscle training.

Questionnaires		Baseline assessment (n=156)	Post-treatment assessment (n=156)	p-value
QUID TOTAL score (0–15), mean (SD)		3.3 (1.3)	2.5 (1.3)	<0.001
	SUI	7.6 (3.7)	5.8 (3.4)	<0.001
	OAB	7 (4)	5.9 (3.7)	<0.001
ICIQ-SF score (0–21), mean (SD)		14.4 (4)	12.1 (4.7)	<0.001
ICIQ-OAB score (0–16), mean (SD)		7.3 (3.7)	6.3 (3.7)	<0.001
I-QOL TOTAL score (22–110), mean (SD)		51.8 (23.7)	58.4 (23.5)	<0.001
	Avoidance or limiting behaviors	50.1 (23.1)	57.8 (22.8)	<0.001
	Psychological impacts	61.7 (27.3)	66.5 (25.9)	<0.001
	Social embarrassment	36.9 (24.9)	42.2 (25.6)	<0.001

QUID: Questionnaire for Urinary Incontinence Diagnosis; SUI: Stress Urinary Incontinence; OAB: overactive bladder; SD: standard deviation; ICIQ-SF: International Consultation on Incontinence Questionnaire-Short Form; ICIQ-OAB: International Consultation on Incontinence Questionnaire-Overactive Bladder; I-QOL: Incontinence Quality of life Questionnaire; Wilcoxon test.

In terms of exercise adherence, women reported engaging in the prescribed exercises with satisfactory frequency ("always," "almost always," and "many times") 74.3% of the time. Remarkably, the PFMT regimen guided by the Diário Saúde^®^ app demonstrated robust acceptance, with only 2% of participants reporting noncompliance with the exercises. Additionally, over 62% of women reported either complete alleviation or a notable improvement in symptoms post-treatment, with an average satisfaction rating of 7.4 on a scale of 0–10.

A post-hoc power analysis was conducted, comparing score values (QUID, ICIQ-SF, and I-QOL) before and after the intervention, based on data from 156 women with UI and utilizing mean and standard deviation values from both assessment points. The analysis revealed a high level of statistical power, ranging from 98.3 to 99.9%, for the comparison of questionnaire scores pre- and post-intervention.

## DISCUSSION

Our findings showed improvements in SUI symptoms, urinary storage symptoms (such as urinary frequency, nocturia, urinary urgency, and urge incontinence), and quality of life. The utilization of the Diário Saúde^®^ app exhibited high levels of adherence, complemented by significantly positive feedback from the participants.

Among various physiotherapeutic modalities, PFMT requires consistent execution and sustained efforts by the patient within a home setting. Notably, our study's outcomes align with the findings of Barret et al.^15^ indicating a preference for app-based guidance over traditional verbal or written instructions for PFMT.

Employing app-guided PFMT holds distinct advantages, primarily stemming from cost reduction compared to in-person sessions due to the elimination of transportation expenses and material resources. Moreover, it facilitates heightened adherence by accommodating flexible timing and location for therapy, further reinforced by reminders and alarms to minimize forgetfulness. Our study revealed an encouraging 74.3% adherence rate among participants who reported regularly performing exercises through the app, reflecting substantial acceptance and compliance with the app's guidance. This level of adherence surpassed that reported in previous studies, indicating the app's efficacy in promoting consistency^15,16^.

During the COVID-19 pandemic, the utilization of the Diário Saúde^®^ app for PFMT exhibited efficacy comparable to in-person programs. Notably, a Cochrane review demonstrated that women undergoing PFMT reported significant improvement, with a success rate of 55% in symptom relief or improvement, compared to 3.2% among untreated individuals^17^. Our study demonstrated a similar success rate of 62.2%, consistent with the outcomes from other mobile app-based interventions^18^.

Our findings emphasized marked improvement in UI symptoms, as indicated by QUID and ICIQ-SF scores, along with enhanced condition-specific quality of life. These outcomes mirror those observed in supervised or unsupervised PFMT interventions^19,20^ and align with a systematic review involving unsupervised behavioral and PFMT programs^21^. A previous randomized controlled trial focusing on a UI app similarly demonstrated enhanced ICIQ-SF scores and quality of life after 3 months of treatment^22^.

Notably, our study documented a significant amelioration of storage symptoms, consistent with the potential of PFMT to mitigate such symptoms^23^. Pelvic floor contractions play a pivotal role in reducing detrusor pressure, elevating urethral pressure, and suppressing the micturition reflex. Our results substantiate the effectiveness of the home-based app-guided exercise regimen in enhancing storage symptoms.

Remarkably, a quarter of our study population tested positive for COVID-19, with half of these individuals reporting exacerbation of UI symptoms. While respiratory symptoms have been the primary focus of COVID-19, it is worth noting that the noticeable increase in abdominal pressure resulting from heightened respiratory efforts, coughing, sneezing, and overall weakness could potentially lead to added strain on the pelvic floor that contributes to the worsening of UI.

Strengths of our study include its pioneering approach in evaluating UI treatment through an app during the COVID-19 pandemic, ensuring continued care and access to health services within the prevailing limitations. The app's incorporation of reminder notifications substantially contributed to participant adherence, a pivotal factor in achieving positive outcomes. Despite the relatively short study duration, the rate of loss to follow-up remained negligible.

The limitation of the study was the absence of physical assessment for pelvic floor muscles, necessitated by pandemic restrictions. Furthermore, the lack of an extended follow-up hinders our understanding of the long-term effects of the treatment. Additionally, the absence of a control group limits the scope of comparison. Future studies should consider control groups, extended follow-up, and face-to-face assessments for a comprehensive evaluation of UI treatment. Despite these limitations, our study demonstrates the feasibility and efficacy of treating UI using the Diário Saúde^®^ app, particularly in the face of pandemic-induced challenges. This approach holds promise for enhancing access to conservative treatment and fostering adherence to PFMT.

**Table 3:** Adherence, subjective improvement and satisfaction of women (n=156) with urinary incontinence treatment.

Adherence treatment, n (%)		Total (n=156)
	Always or almost always	57 (36.5)
	Many times	59 (37.8)
	Same times	29 (18.6)
	Few times	8 (5)
	Never or almost never	3 (1.9)
Subjective improvement		n (%)
	Cured	16 (10.2)
	Almost cured	76 (52)
	Same symptoms as before treatment	49 (31.4)
	A little worse	15 (9.6)
	A lot worse	0
Self-reported* satisfaction (0–10)		Mean (SD)
		7.5 (2.5)

UI: urinary incontinence; app: mobile application; PFMT: pelvic floor muscle training; N: absolute frequency; SD: standard deviation;

* scale from 0 to 10.

## CONCLUSION

In light of the challenges posed by the COVID-19 pandemic, the implementation of a home-based mobile app program has proven to be successful in reducing symptoms of SUI. This innovative strategy not only resulted in significant enhancements in both quality of life and storage symptoms but also exhibited remarkable levels of adherence and participant satisfaction with the treatment.
